# Unobtrusive Sensing at Home Towards Healthcare 5.0: Technologies, Applications, and Future Directions

**DOI:** 10.3390/bios16050250

**Published:** 2026-04-29

**Authors:** Regina Oliveira, Joana Simões, Pedro Correia, António Teixeira, Florinda Costa, Cátia Leitão, Ana Luísa Silva

**Affiliations:** 1 Institute for Nanostructures, Nanomodelling and Nanofabrication (i3N—Aveiro), Department of Physics, University of Aveiro, 3810-193 Aveiro, Portugal; regina.oliveira@ua.pt (R.O.); joanasimoes02@ua.pt (J.S.); pmcorreia@ua.pt (P.C.); flor@ua.pt (F.C.); catia.leitao@ua.pt (C.L.); 2 Institute of Electronics and Informatics Engineering of Aveiro (IEETA), Department of Electronics, Telecomunications & Informatics, University of Aveiro, 3810-193 Aveiro, Portugal; ajst@ua.pt

**Keywords:** health parameters, Healthcare 5.0, home health monitoring, unobtrusive sensors, vital signs

## Abstract

The growing prevalence of chronic diseases, population aging, and the shift toward preventive and personalized care under Healthcare 5.0 have increased the need for continuous health monitoring beyond clinical settings. While wearable devices enable remote monitoring, their long-term use is often limited by user compliance, comfort issues, battery dependence, and disruption of daily routines. To address these limitations, unobtrusive home-based health monitoring systems have emerged, integrating sensing technologies into domestic environments and everyday objects. This review provides a system-level analysis of unobtrusive health monitoring technologies for smart homes. It examines seven major sensing approaches, including camera-, laser-, radar-, infrared-, mechanical-, bioelectrical-, and optical-based sensors, and their integration into four home environments: living areas, bathrooms, bedrooms, and home offices. For each sensing modality, the operating principles, monitored physiological parameters, representative applications, and key advantages and limitations are discussed. Overall, existing solutions reveal trade-offs among measurement accuracy, robustness in real home conditions, energy autonomy, privacy preservation, and user acceptance. Heart rate and respiratory rate are the most commonly monitored parameters, while multimodal and clinically validated systems remain limited. Although unobtrusive sensing technologies show strong potential for proactive and personalized healthcare, challenges related to accuracy, interoperability, privacy, and cost continue to hinder large-scale adoption.

## 1. Introduction

The healthcare landscape is changing rapidly, undergoing a profound transformation, driven mainly by social changes, individuals’ expectations, and new demands, and by the technological innovations of the modern age. At its forefront is the paradigm of Healthcare 5.0, the latest evolution in healthcare that focuses on a patient-centered approach while integrating advanced technologies like artificial intelligence (AI), internet of things (IoT), robotics, blockchain, and 5G. Healthcare 5.0 is driving continuous and imperceptible monitoring of health parameters within our daily lives, particularly at home, offering unprecedented opportunities for preventive and proactive health management and personalized medicine [1,2].

Home-based health monitoring plays a key role in this transformation, as it shifts healthcare delivery from sporadic clinical interactions to continuous observation within daily life. Traditional monitoring approaches rely mostly on wearable devices, which have demonstrated clear benefits for remote health assessment. However, long-term use of these devices is often limited by factors such as user compliance, comfort, battery maintenance, and interference with daily routine [3,4]. These constraints have motivated increasing interest in unobtrusive and invisible health monitoring, an approach in which sensing technologies are embedded into the living environment itself rather than attached to the body.

Unobtrusive home-based monitoring makes use of sensors that are integrated into everyday objects, furniture, and building infrastructure to enable continuous measurement of physiological parameters without requiring active user action. By operating passively in the background, these systems support preventive healthcare, early detection of anomalies, and long-term monitoring without disrupting the user’s daily routine [5]. A wide range of sensing approaches has been explored for this purpose, including camera-based, laser-based, radar-based, infrared, mechanical, bioelectrical, and optical sensors, each of them offering distinct capabilities for vital signs monitoring.

In recent years, several review articles have addressed aspects of home-based or contactless health monitoring [5]. Existing works often focus on specific sensing modalities, such as camera-based [6,7] or radar-based systems [8], applications like sleep monitoring [9], or wearable and nearable technologies [10]. While these reviews provide valuable insights within their specific scopes, they offer a limited perspective on how heterogeneous sensing technologies can be systematically integrated into home environments. Consequently, some aspects like the deployment of sensing technologies across different rooms, interaction with household objects, privacy considerations, and cross-technology trade-offs are not always analyzed.

To address these gaps, this review provides a comprehensive and unified analysis of unobtrusive health monitoring technologies for smart-home environments. Unlike prior works, the operating principles underlying different vital sign measurement approaches are explored, as well as their integration into everyday household objects and furniture, and deployment across different home rooms, including living areas, bedrooms, bathrooms, and home offices. By organizing the literature according to both sensing modality and room, this review offers a system-level perspective on how unobtrusive monitoring can effectively be employed in our homes.

In addition, this work synthesizes recent research findings and technological advancements from commercial systems already available on the market to ongoing research into emerging technologies. This review aims to provide a broader view of current capabilities, limitations, and technology availability. Through a comparative analysis of strengths, limitations, and application contexts, this review highlights some trade-offs related to sensing accuracy, robustness to motion and environmental variability, privacy, and user acceptability. Finally, open challenges and future research directions are discussed in the context of Healthcare 5.0, emphasizing the importance of unobtrusive monitoring in enabling proactive, personalized, and accessible healthcare.

In this review, the term unobtrusive health monitoring refers to approaches that measure physiological or behavioral parameters with minimal disruption to daily life, without requiring deliberate user action or sustained compliance, and without the need to wear or actively manage dedicated devices. Within this context, passive monitoring denotes sensing that occurs automatically without active user participation or intentional triggering. The term invisible monitoring is used more narrowly to describe sensors that are physically embedded, concealed, or otherwise integrated into the environment, where the user cannot see them, being unaware of their presence during health monitoring. While passive or contactless systems may be invisible, invisibility is not a necessary condition for a system to be considered unobtrusive. These distinctions are adopted throughout this review to ensure consistent terminology when describing unobtrusive sensing technologies.

Accordingly, the principles and techniques of unobtrusive health monitoring are reviewed in Section 2. Different home-based objects and used cases for continuous and unobtrusive health monitoring systems are described in Section 3, particularly for living spaces, bathroom, bedroom, and office. All the monitoring technologies are summarized in Table A1. The presented overview ends with the advantages and challenges of health monitoring in daily life without disrupting routine activities. A reflection on the future directions and opportunities is also presented.

### Aims and Contributions

This review aims to provide a comprehensive and system-level overview of unobtrusive health monitoring technologies for domestic environments. The main contributions are:

- System-level summary of unobtrusive health monitoring technologies, covering camera-based, radar-based, infrared, mechanical, bioelectrical, and optical sensing approaches;
- A room- and object-based taxonomy that categorizes existing solutions according to their integration into different domestic environments, including living spaces, bedrooms, bathrooms, offices, and shared household infrastructure;
- Revision of academic and commercial solutions, combining recent research prototypes with commercially available smart-home health monitoring systems;
- Identification of research gaps and future directions, with emphasis on scalability, interoperability, privacy-aware development, energy autonomy, and alignment with the Healthcare 5.0 paradigm.

## 2. Monitoring Technologies

**Figure 1:**
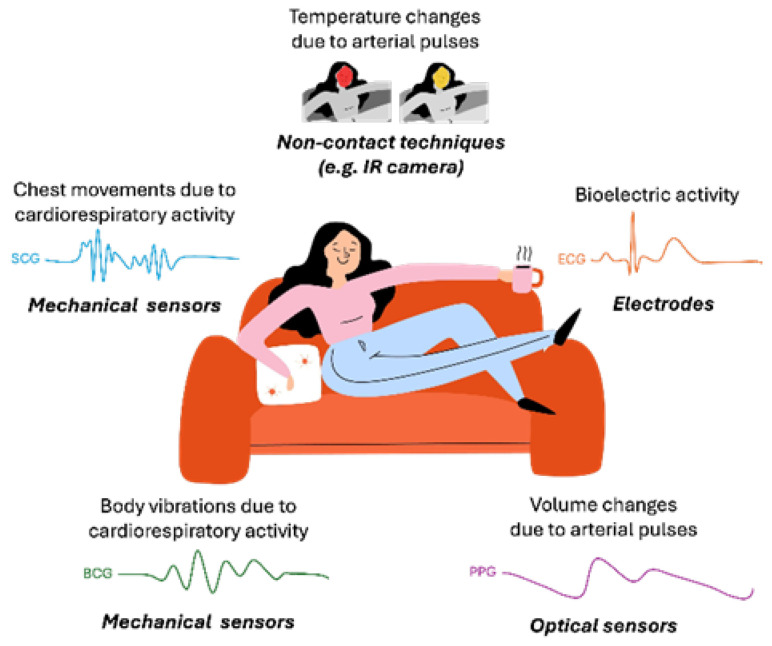
Schematics of the biosignals of a normal subject resting, with examples of sensors used in invisible health monitoring (IR—infrared; ECG—electrocardiography; BCG—balistocardiography; PPG—photoplethysmography; SCG—seismocardiography).

Invisible sensing of health parameters involves the use of technologies that allow for monitoring and measuring physiological and biological signals without the need for user action. They offer unobtrusive and continuous monitoring, which is especially useful in settings like remote patient care and smart environments. On one side, there are fully contactless monitoring technologies, such as camera, radar-, infrared-, or laser-based sensing, which operate without physical interaction with the user. On the other hand, there are contact-based approaches, in which sensors are embedded into everyday objects (e.g., beds, chairs, toilets, and floor surfaces) that users naturally interact with. For example, during daily activities, vital signs suffer alterations that can be measured by sensors embedded in the environment (e.g., couch), as schematized in Figure 1, where contact and non-contact technologies are represented for invisible health sensing. The type of biosignals that can be obtained by each technique is also represented and will be detailed below.

### 2.1. Non-Contact Methods

#### 2.1.1. Camera-Based

Camera-based technologies use imaging equipment to unobtrusively monitor health parameters. These systems are affordable, widely accessible, and have shown significant advances and potential applications in smart homes and telemedicine. The field of camera-based technologies comprises various types of cameras, each tailored to specific applications and functionalities, namely, red–green–blue (RGB, wavelength: 350–740 nm), near-infrared (NIR: 740–1000 nm), and far-infrared (FIR: >1000 nm) cameras [6,7,11].

RGB cameras are commonly used to monitor vital signs such as heart rate (HR), respiratory rate (RR), oxygen saturation (SpO_2_), and blood pressure (BP). They detect subtle color changes or motion caused by physiological activities, such as blood flow and breathing. From the variations in the pixels’ intensity, it is possible to derive these health parameters. NIR cameras are used to measure HR and RR through the detection of subtle temperature variations and surface movements.

NIR cameras measure HR and RR similarly to RGB cameras but have the capability of operating in low-light or dark environments. This is particularly beneficial for applications like sleep monitoring and analysis. However, NIR cameras have reduced absorption by hemoglobin and a lower signal-to-noise ratio (SNR). Optical filtering at the acquisition stage can improve spectral selectivity and signal quality. After region-of-interest segmentation, temporal filtering, normalization, and component decomposition are typically applied to suppress noise and motion artifacts. In addition, machine learning and deep learning models have shown the ability to extract subtle spatiotemporal physiological patterns from low-SNR NIR image sequences, improving the robustness of vital sign estimation [12].

FIR cameras, also known as thermal cameras, are used to monitor skin temperature by detecting the infrared radiation emitted from the body. FIR cameras can monitor HR and RR by detecting periodic movements. Additionally, FIR cameras are ideal for nighttime applications such as sleep studies, neonatal care, or intensive care unit monitoring. They perform well under varying illumination conditions, including darkness, and address privacy concerns since they do not capture identifiable visual data. However, FIR cameras are typically top-tier products and come with a higher cost compared to other camera types [6,7]. 

Although many advances have already been made in this field, some challenges remain, namely, the ones related to hardware, motion artifacts, and variability in natural settings. Further developments are needed to ensure robustness in dynamic settings, cope with movement, facial expressions, varying illumination, and different camera–subject distances. Camera-based systems also increase privacy concerns, since individuals tend to prefer coarse-grained silhouettes from depth sensors over fine-grained images/videos from regular RGB cameras. Privacy-friendly hardware choices are essential for user acceptance [6].

#### 2.1.2. Laser-Based

Laser-based techniques have been explored for continuous and unobtrusive monitoring of vital signs, with the advantage of being able to penetrate some media, like clothes and wood, to remotely sense physiological parameters. Laser Doppler vibrometry is a promising technique for contactless heartbeat detection, as it can measure the vibrations of the skin caused by the heartbeats and arterial pulses, particularly over major arteries like the carotid [13]. Nonetheless, laser-based methods face challenges like motion artifacts, variability between subjects due to differences in physiology, and environmental noise like thermal distortion from sweating and air flow. More robust algorithms, using learning-based approaches, and controlled conditions are needed to mitigate these issues and to improve reliability and robustness in real-world settings [13].

#### 2.1.3. Radar-Based

A radar-based system is designed to estimate the frontal distance and/or the velocity of objects (i.e., targets) able to reflect the electromagnetic waves they generate. A radar system consists of a transmitter, producing radio waves with known properties and radiating them along a predetermined direction using a single or multiples antennas, and the receiver captures the waves reflected by the targets [14]. For the case of vital signs monitoring, such as RR and HR, the chest wall is the target, as periodic vibration information resulting from respiration and heartbeat modulates the reflected electromagnetic wave [15]. The radar-based systems can be classified based on their wavelength, which can be of a few centimeters, and millimeter-wave (mm-wave) radars (≥30 GHz). They can also be classified as short-range (able to measure a maximum range of about 30 m); medium-range (up to about 100 m); and long-range radars (maximum range in order of 250 m) [14]. Finally, depending on the type of signal they transmit, radars can be of continuous wave (CW), ultra-wideband (UWB), or frequency-modulated continuous wave (FMCW) [8,16].

The CW radars are the most common type due to their simplicity. A transceiver sends a single-frequency continuous-wave signal towards the moving chest of the subject through the transmitter antenna. The reflected wave is detected by a receiver antenna, and the chest displacement due to heartbeat and breathing motions results in a variation in the received signal phase [14]. This signal is demodulated and processed to obtain the RR and HR [8]. CW radars have the advantage of a simple radio structure and low-power consumption. However, reflections from other moving objects or people can easily contaminate echoes from the target [15,16].

UWB radars are advanced systems that use short pulses of electromagnetic waves to detect vital signs such as RR and HR. They transmit ultra-short pulses and analyze the reflected signals to measure chest movements caused by breathing and heartbeat. UWB radars excel in detecting tiny movements with high-resolution, making them ideal for unobtrusive monitoring. UWB offers superior resolution and can operate effectively in cluttered environments or through obstacles like walls. However, this type of radar faces challenges such as signal attenuation over long distances, interference from environmental noise, and difficulty isolating heartbeat signals due to overlapping breathing movements [16,17,18].

FMCW radars are increasingly used for non-contact monitoring of vital signs, such as RR and HR. The FMCW radar continuously emits a radio signal whose frequency gradually increases and decreases over time. When this signal hits a subject, it reflects back to the radar. By comparing the transmitted signal with the reflected one, the radar determines the distance to the subject and detects displacements caused by breathing and heartbeat, which are embedded in the phase of the reflected signal. Advanced signal processing techniques are applied to isolate RR and HR data [8,11]. Compared to other types of radars, FMCW can distinguish between multiple targets, and unlike the UWB radar, FMCW requires less complex electronics, making it more cost-effective and energy-efficient. However, challenges such as susceptibility to interference from other devices and the need for advanced signal processing to isolate vital signs still exist [8,16].

#### 2.1.4. Infrared Sensors

Pyroelectric infrared (PIR) sensors measure changes in the amount of IR radiation (blackbody radiation) emitted by moving subjects, which vary according to the temperature and surface characteristics of objects in front of the sensor. PIR sensors have several advantages for invisible detection applications, since they are low-power consumption, have a wide detection area, and are affordable and reliable. These sensors are commonly used for motion detection, but they can also measure vital signs such as RR and HR by detecting periodic temperature fluctuations caused by chest movements during breathing or blood flow dynamics. However, these sensors face challenges such as sensitivity to environmental temperature changes, interference from unrelated movements, and their reliance on a clear line of sight, which can be blocked by obstacles [19].

To mitigate the inherent low spatial resolution of PIR sensors, most approaches exploit the temporal characteristics of the acquired signals rather than spatial features. Physiological activities such as respiration and cardiac motion induce periodic thermal and movement patterns that can be extracted using band-pass filtering, adaptive filtering, autocorrelation techniques, and frequency-domain analysis. Furthermore, the deployment of multiple PIR sensors or small sensor arrays can enhance effective spatial discrimination and improve robustness against environmental interference and unrelated motion [19].

### 2.2. Contact Methods

#### 2.2.1. Mechanical Sensors

Mechanical sensors are devices designed to detect and measure physical changes in the environment, such as force, pressure, strain, displacement, and vibration. The key types of mechanical sensors include force and pressure sensors, which measure applied load, and deformation sensors and displacement sensors that measure movement or position, among others [20].

According to the transducing method, they can also be of different natures, namely, capacitive, piezoelectric, piezoresistive, and triboelectric. Though these sensors have different transducing mechanisms, they share a similar structure, typically comprising electrodes and active sensing components. In each type, the active sensing component responds to strain or pressure, leading to a change in resistance, capacitance, or induced charge, which in turn alters the electrical signal output proportional to the measurand. While resistive and capacitive sensors require an external power source, piezoelectric and triboelectric sensors can directly convert pressure or strain into an electrical signal, enabling self-powered sensing [20].

For instance, capacitive sensors can detect the subtle chest movements associated with breathing or the heart’s pulsations, translating these movements into readable signals. These sensors do not require direct skin contact, as they rely on indirect detection of movements, making them more suitable for non-intrusive continuous monitoring [21].

Also, in the scope of mechanical sensing, inertial measurement units, such as accelerometers and gyroscopes, allow for measuring motion and orientation. This data has a crucial role in inertial sensing, being used in several applications, such as monitoring physical activity, sleep patterns, gait, neurological disorders, and balance, among others [22].

Mechanical sensors have been used in a very wide range of applications, from load detection to vibrations, such as those caused by the cardiorespiratory system. Measurements of heart-induced motions, including displacement, velocity, and acceleration, can be performed in two ways: ballistocardiography (BCG), which is the measurement of whole-body recoil forces in response to cardiac ejection, and seismocardiography (SCG), relative to the local chest surface measurement of cardiac-induced vibrations [23]. Despite their wide applicability, they face several limitations and challenges that can affect their performance and reliability, namely, their sensitivity to external factors such as temperature changes, humidity, and vibrations, which can introduce noise or errors in measurements. Additionally, prolonged use in harsh environments may lead to wear and tear of mechanical components, impacting durability and lifespan [24].

#### 2.2.2. Bioelectric Sensors

Bioelectrical measurements involve detecting and analyzing the electrical signals generated by physiological processes. Techniques like electrocardiography (ECG), electroencephalography (EEG), and electromyography (EMG) are used to record the electrical activity of the heart, brain, and muscles, respectively. Bioelectrical sensors, despite their significant potential in healthcare, face several limitations and challenges. One major issue is their durability, as these sensors can degrade over time due to exposure to harsh environmental or biological conditions. They are also susceptible to interference from external electrical signals or devices, which can lead to inaccurate readings [24,25].

#### 2.2.3. Optical Sensors

An optical sensor is a device that can monitor a variable by tracking changes in the received light signal. Those changes can be phase, intensity, and polarization, among others [26].

Photoplethysmography (PPG) is one of the most known optical sensing technologies for biomedical monitoring [27]. It employs one or several light sources (usually LEDs) to emit light into tissue, and photodetectors to capture the light that is either reflected or transmitted through the tissue. This method is widely employed to measure various vital signs, including SpO_2_, HR, RR, and BP. The signal obtained through PPG reflects the pulsatile changes in blood volume within the peripheral microvasculature, driven by the pressure pulse of each cardiac cycle [28]. Traditionally, the sensing unit is placed in direct contact with the skin; therefore, it is here specified as a contact technique. However, studies have demonstrated non-contact PPG reliability in monitoring HR [29].

Moreover, other optical sensors, such as optical fiber-based, can also be used to detect strain and pressure, and therefore perform body monitoring and health supervision in a mechanical way [30].

Optical sensors face several limitations and challenges that impact their performance and widespread adoption. Motion artifacts and environmental factors such as ambient light, pressure, corrosion, or low-current speeds are the main challenges that affect optical sensors’ accuracy. Also, the need for robust signal processing algorithms further complicates their integration into various applications where processing power is limited. Additionally, optical sensors, especially fiber optic types, can be fragile and require careful handling during installation and maintenance [24].

## 3. Applications of Unobtrusive Health Monitoring in Domestic Environments

**Figure 2:**
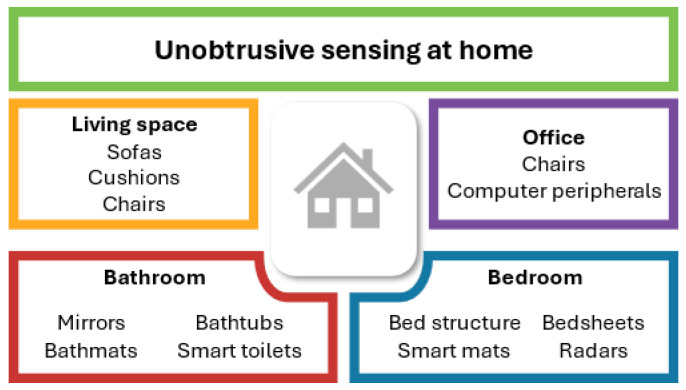
Furniture and accessories can integrate sensor systems for continuous invisible health monitoring in the different rooms of the house.

The growing demand for remote health monitoring has led to the design of innovative technologies, enabling unobtrusive vital signs monitoring at home. By unobtrusively tracking physiological parameters, these systems empower users to take an active role in their health, without the need for them to wear any monitoring devices or change their daily routine. The analysis of the collected vital signs can bring valuable information about the user’s health status, allowing early diagnosis or the follow-up of some diagnosed patients [5]. These systems can be spread around the house, like intelligent floor tiles [31] and through walls radars [18,32], or be specific for one room and object, like smart toilets [33,34,35,36,37,38,39,40] or sensorized bed sheets [41,42,43,44]. The next subsections highlight these innovative ideas, categorized by their main use in various rooms of the house, as illustrated in Figure 2.

### 3.1. General Household Monitoring

Smart systems can be spread throughout the house through objects that can be in any room, monitoring users’ activities, like floor tiles [31,45] and wireless devices [18,32,46,47,48].

#### 3.1.1. Floor Tiles

Sensors can be embedded in the house’s floor to obtain health information from individuals. Chang et al. [31] developed, in 2018, smart floor tiles capable of monitoring HR across various household settings. These innovative tiles offer a versatile solution by measuring HR in both standing and sitting positions. When the user is seated, the tiles collect ECG data, while in a standing position, they capture BCG. The determination of whether the user is standing or seated is made by analyzing the contact force and pressure exerted on the smart floor tile. Each tile design consists of four load cells positioned at the bottom of a plexiglass platform, and four stainless steel electrodes placed on the top surface. This configuration ensures precise and reliable measurement of HR regardless of the user’s posture. The integration of these smart floor tiles into household compartments enables seamless and continuous monitoring of HR throughout daily activities, providing valuable insights into cardiovascular health and overall well-being.

Floor tiles are also capable of giving information about human presence, individual identity, and individual activity, and can also be used for eventual fall detection [45].

#### 3.1.2. Wireless Monitoring

Wireless technologies are an emerging tool for health monitoring at home. Most of these technologies emit a wave with a known frequency and detect the changing of the wave’s phase caused by the body’s natural vibrations induced by the heartbeat and the respiratory motion.

Li et al. [32] and Xie et al. [18] developed through-wall monitoring systems based on an UWB radar in 2021 and 2022 respectively. The first group uses a multiple-input multiple-output (MIMO) imaging radar to measure the vital signs of multiple subjects by extracting the radar image’s phase. These radars present an advantage when compared to CW ones, since they also give information about subjects’ location [32]. The second research group used a pair of UWB radars combined with a depth camera, calling it VitalHub. This system is able to distinguish between cohabiting subjects, associating the vital signs to each one, and generate a context of the subjects’ activities at home due to the presence of a depth camera in it, increasing the robustness and reliability of the system [18].

The house wall’s surface can also be modified to measure vital signs. Kazim et al. presented, in 2023 [47], a prototype of a reconfigurable intelligent surface (RIS) for vital signs detection. This surface can be employed in the house’s walls, and consists of several reflecting components, developed for 5G communications. The system can distinguish the incoming beams from multiple directions in that frequency band. Due to the reflective property of the RIS, the user does not need to be in front of the signal’s receiver for vital signs detection, allowing the measurement of these signals in any room’s site. The tests performed by this research group showed the viability of this system to detect the users’ HR and RR.

More recently, NASA’s space technology has been adapted for home health monitoring through a radar-based system. A wall-mounted device uses low-power radio waves and AI-driven signal processing to detect HR, RR, and stress from up to approximately three meters away [46,48]. Now commercialized by Advanced TeleSensors (Austin, TX, USA) as the Cardi/o Monitor, it offers a touchless solution that transmits real-time health data to a smartphone app, making it ideal for eldercare and chronic condition management. With emissions far lower than Wi-Fi and no need for batteries, the system ensures safety, user-friendliness, and continuous monitoring, illustrating how space innovations can enhance proactive care at home [46,48].

### 3.2. Living Room and Common Areas

Living spaces in a house, such as the kitchen and the living room, are areas where people gather and share time together. This way, in these areas, certain furniture and accessories can incorporate sensors capable of detecting users’ vital signs, such as load cells [49,50] and capacitive coupling electrodes [51]. For instance, a smart cushion on a chair or a sensorized sofa can monitor health metrics while users relax, sit, or lie down.

#### 3.2.1. Cushions

Cushions are home accessories that can be present in several rooms.

In 2019, Malik et al. presented a smart seat cushion as a novel system for HR monitoring through BCG signal acquisition. The cushion is equipped with load cells to obtain the BCG signal that is then processed to calculate the user’s HR. This prototype was developed by modifying an ObusForme Seat cushion and consists of three layers: (1) a stainless steel metallic plate at the bottom, (2) a modified bathroom scale with four strain gauge–type load cells placed on top of the plate, and (3) a polyurethane foam to enclose the plate and scale. The solution presented is portable, affordable, unobtrusive, and can be easily integrated into a home-based environment for zero-effort HR monitoring. The cushion ensures that users can monitor their HR comfortably and conveniently in a seated position while relaxing or eating in the kitchen [49,50].

Besides monitoring vital signs, smart cushions can also identify the subject through the BCG signal’s pattern analysis. Zhang et al. developed a prototype that uses a microbend fiber sensor, embedded in a cushion, to detect the BCG signal of who is leaning back on the cushion. When the user leans back on this cushion, it is identified while the heart rate, respiration, and body motion are monitored [52].

#### 3.2.2. Sofas

In the living room, the most imposing piece of furniture is the sofa. For instance, the company H&T (Shenzhen, China) has upgraded the traditional sofa by introducing several functionalities for health monitoring, position and angle monitoring and adjustment, firmness control and changing through AI analysis, lumbar support, and massage. For health monitoring, sensors have been integrated for HR and RR assessment. However, the company does not share information about the sensing technologies applied [53].

#### 3.2.3. Chairs

Zheng and Tang [51] presented a chair with embedded capacitive coupling electrodes for ECG measurement. The backrest of the chair was fully covered with three flexible electrodes, composed of a conductive silver fabric, foam, and an insulation material. Two electrodes were placed side by side to cover the thoracic region, while the third electrode covered the entire waist area in the chair. Through amplification circuits and signal filtering and processing, the researchers obtained ECG signals pretty similar to the ones obtained with contact ECG, and were able to extract the HR and HR variability (HRV).

### 3.3. Bathroom Smart Monitoring Solutions

The bathroom of a house is also an important space for personal health care. It is a space where most people start and end each day, which has led to the expansion of the existing functions of this space. The main role of the bathroom is no longer only associated with cleanliness and personal hygiene, as it is also beginning to be linked with preventive medicine [54].

Factors such as social health awareness, increasing concern about personal health, and the need to monitor it are shaping current trends in the design of domestic bathrooms and their elements [54].

Some of the elements of a bathroom that have been used to monitor people’s health are presented below in detail: mirrors, mats, bathtubs, and smart toilets. These bathroom’s appliances monitor the vital signs through the analysis of data collected from cameras (remote PPG and thermal imaging) [55,56,57,58,59,60], load cells (BCG) [61], capacitive coupling and stainless steel electrodes (ECG) [33,39,62,63], or/and optical sensors (PPG) [36].

#### 3.3.1. Mirrors

A smart mirror combines sensors and the ability to interact with the user, collecting and transmitting information, mainly by using integrated displays. In recent years, smart mirrors have been applied in different contexts such as the automotive industry, fashion, and sports. A smart mirror can recognize the user’s voice and face and allows the display of general information such as weather, time, date, traffic, news, reminders, and notifications, among others [54,57,58].

**Figure 3:**
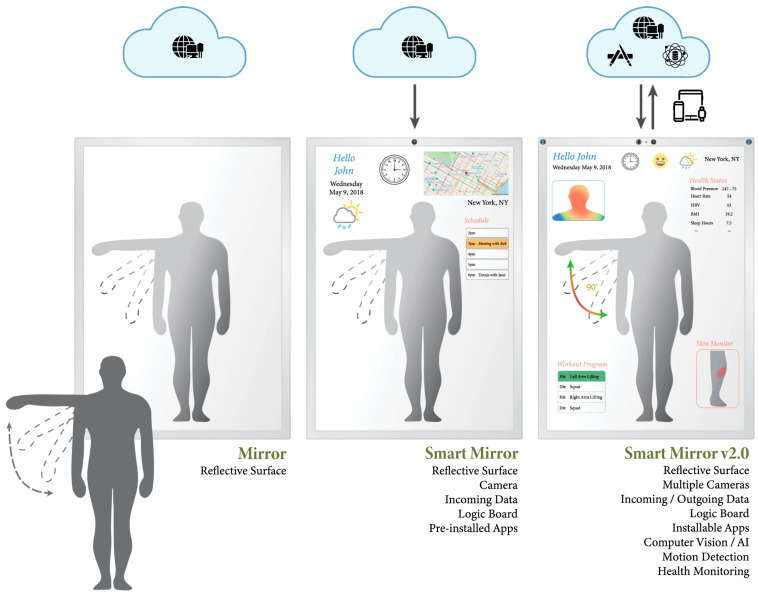
Evolution of mirrors from simple reflective surfaces to interconnected, cloud-based smart systems, integrating activity tracking, passive health and motion monitoring, contextual data, and digital health biomarkers [58]. Figure licensed under a Creative Commons Attribution 4.0 International License (CC BY 4.0).

Recent research has led to the emergence of a second generation of smart mirrors with applications in the healthcare field. These are part of a growing set of technologies that make it possible to monitor health and collect relevant information from individuals in a non-contact way, outside medical facilities, and during their routine activities [54,58]. To achieve this, as presented in Figure 3, cameras and IR sensors are integrated in the mirror to assess various metrics, such as HR, body temperature (BT), body posture, changes in skin characteristics (e.g., color and texture), and emotional states [57,58]. All these parameters are meaningful health markers, and their monitoring at home promises more accurate and useful diagnoses.

Soppimath et al. proposed the Medical Mirror, an interactive interface that uses a thermal imaging system to provide real-time estimates of the user’s HR without the need for external sensors. This is achieved by using computer vision and signal processing techniques on the acquired optical signal, reflected on the user’s face [58,60].

Currently, there are several commercial smart mirrors available, including the Anura MagicMirror from the digital health company NuraLogix from Toronto, Canada [59] and the Artemis and Themis from CareOS, France [56].

The Anura MagicMirror is a smart mirror that analyses facial blood flow through a video camera to estimate vital signs and disease risk assessments. It provides information like BP, HR, body mass index, mental stress, heart attack risk, cardiovascular disease risk, and many others, by simply sitting down in front of the mirror. Larger screen devices also target industry partners like retailers, gyms, schools, corporations, and healthcare facilities to create proactive health solutions for employees, customers, and patients [59].

Smart mirrors from CareOS are equipped with various sensors such as RGB cameras, temperature sensors, and UV light sensors, with the purpose of monitoring users’ skin, hygiene, and mental health. Initially, the mirrors developed were only capable of measuring BT. In 2022, CareOS partnered with Binah.ai, a provider of general health and wellness video-based monitoring solutions powered by AI, to take health monitoring capabilities to the next level with smart mirrors. Binah.ai’s technology provides additional health data, including BP, HR, SpO_2_, RR, sympathetic stress, parasympathetic activity, and pulse–respiration quotient [55,56].

#### 3.3.2. Bathmats

For many people, stepping on the scale can trigger a range of emotions. Some might feel excited to see progress, while others might feel nervous or frustrated. Although it is important to keep track of weight, seeing small changes every day can sometimes be discouraging to step on the scale. A smart bathmat appears as a new, discrete tool for improving and monitoring health. Bathmats can embed sensors (load cells) for weight estimation, making it easy to take measurements without the awkwardness of using a conventional scale. Users can simply step onto their bathmat as they get out of the shower or while they brush their teeth, and the measurements are automatically taken.

Baracoda (France) company presented, in 2020, the world’s first smart bathmat, Bbalance. Through integrated sensors and a dedicated app, Bbalance offers an effortless smart system to track users’ health parameters throughout the day, in their bathrooms. Using advanced pressure-mapping technology, the Bbalance bathmat captures a detailed image of the user’s footprint and is able to take daily measurements of posture, weight, balance, and body composition. Users are then presented with a score that ranks their individual balance and postures, which can be improved with guided exercises [61].

#### 3.3.3. Bathtubs

Bathing can represent a risk for individuals with chronic cardiopulmonary diseases, due to the thermal effect and the pressure of the water on the body. Therefore, cardiopulmonary monitoring during bathing is useful for the early detection of abnormal heartbeats and respiratory conditions, as well as for the prevention of drowning caused by a sudden heart attack. Several studies have developed smart bathtubs in which the ECG is measured using electrodes, demonstrating that integrating electrodes into the bathtub is effective for health monitoring [62,63]. However, currently, there are no commercial intelligent bathtubs available.

Motoi et al. [63] designed a new system where the electrodes were placed outside of the bathtub wall, making them invisible to users. To achieve this, a capacitive coupling electrode configuration was designed to be fixed outside the bathtub. The ECG signals and respiration curves were successfully obtained in all the tested subjects. This new system also proves to be superior to previous systems, as the electrodes are applied in a non-contact configuration.

**Figure 4:**
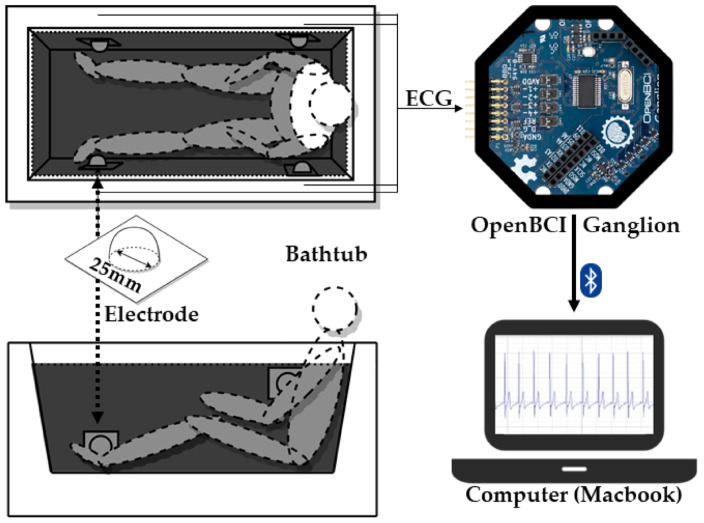
ECG and respiration monitoring in a bathtub [62,63]. Figure licensed under a Creative Commons Attribution 4.0 International License (CC BY 4.0).

In 2022, Li et al. [62] explored an alternative to the exercise stress test (EST) by analyzing HRV during bathing (Figure 4). The developed bathtub had four electrodes attached to the inner wall, at the limb level, and ECG signals from 10 healthy individuals were collected during EST and bathing. During the procedure, the water temperature and bath duration were controlled to match the different stages of the EST. Through this study, they concluded that bathing could serve as a home alternative to EST.

#### 3.3.4. Smart Toilets

**Figure 5:**
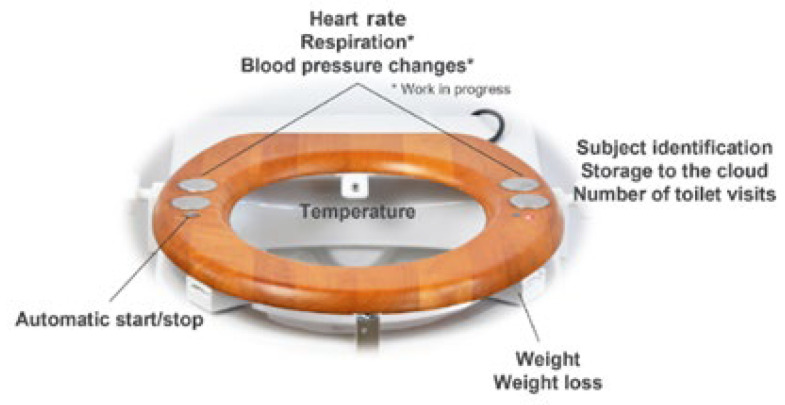
Smart toilet seat [68]. Figure licensed under a Creative Commons Attribution 4.0 International License (CC BY 4.0).

Toilets and toilet seats can also integrate sensors, both to measure vital parameters and/or to collect and analyze biological samples. Smart toilets (as the one presented in Figure 5) can be integrated into daily routines without requiring users to change their habits. This allows for continuous, discreet, and non-intrusive monitoring of various biomarkers that can indicate health deviations [64,65,66]. So far, these smart toilets have been integrating various types of sensors and cameras to measure parameters such as HR, RR, SpO_2_, and BT, as well as to analyze and collect biological samples of urine and/or feces, which provide relevant clinical information for the diagnosis of various diseases and disorders [65,67].

Conn et al. presented a cardiovascular monitoring system based on a toilet seat, focusing on cardiovascular diseases (CVDs). The seat has three stainless steel electrodes for ECG measurement and integrated sensors for measuring the BCG and PPG. This seat can perform clinically relevant measurements of HR, systolic and diastolic BP, stroke volume, weight, and SpO_2_, discreetly. This system, currently called Heart Seat and owned by Casana (Rochester, NY, USA), received FDA approval in 2023 to measure HR and SpO_2_ [33,35].

Also applied to CVDs and considering the need for new non-invasive cardiovascular monitoring methods, the company OLI, S.A. (Aveiro, Portugal), suggested a system for daily ECG monitoring of the user in 2021. This proof-of-concept system consists of a toilet seat that allows passive ECG measurement. To test this new approach, four pairs of dry polymer electrodes with different textures and in different positions were embedded in an experimental seat for ECG signal acquisition. The developed seat allowed the transmission of collected data to a device via Bluetooth. This system has also demonstrated the possibility of measuring ECG signals on the toilet [39].

In 2022, Park et al. [66], with the aim of monitoring COVID-19 non-invasively, developed a prototype of a smart toilet. The prototype included temperature and PPG sensors to measure BT and SpO_2_.

Indeed, currently, there are some commercial smart toilets, such as Çava Seat (Çava Health, Irvine, CA, USA) [34], The Heart Seat (Casana, Rochester, NY, USA) [33], Synsol (Geometry Healthcare, Beijing, China) [40], One Planet (OnePlanet Research Center, Nijmegen and Wageningen, The Netherlands) [38,68], and Haro (Hamberger Medical, Stephanskirchen, Germany) [37].

### 3.4. Sleep and Health Track in the Bedroom

Sleep disorders have a great impact on mental and physical well-being, posing significant risks to overall health, including immune and endocrine system deregulation. Long-term effects can increase the risk of severe conditions such as heart attacks, strokes, hypertension, diabetes, and even mortality [9].

Monitoring vital signs such as RR, HR, and BP, along with sleep positions, has become increasingly necessary because sleep disorders are rising as a global health concern. Sensorized mats, pillows, and bed sheets can conveniently monitor these parameters in the bedroom. Also, wireless devices can be used for the same parameters’ estimation in the bedroom [10,69].

While polysomnography (PSG) remains the gold standard for assessing sleep quality and disorders, its complexity and invasiveness limit its practicality for daily monitoring outside clinical settings. The need for specialized teams during recording restrains its adoption for home healthcare and long-term monitoring purposes [9].

Wearable devices such as smart wristbands, watches, and intelligent clothing present the convenience of real-time sleep monitoring in a more simple and non-invasive way. However, a common limitation of these devices is their need to be close to the skin, which can lead to some discomfort and ultimately impact the quality of an individual’s sleep. Additionally, the accuracy of these devices is often compromised by external factors such as sweat, hair, skin pigment variations, and movement, further constraining their effectiveness for sleep monitoring. These challenges significantly constrain the widespread adoption of wearables for sleep monitoring purposes [9,10].

Technological advancements pave the way for non-intrusive, cost-effective, user-friendly solutions, aiming for real-time monitoring of vital signs, body movement, and sleep position. Smart pillows, mattresses, and bed sheets crafted from intelligent textiles stand out among these systems. These solutions make use of principles such as ultrasonic, optical, and piezoelectric technology. Some of these systems, while promising, present certain limitations, such as reduced accuracy, low sampling rates, poor flexibility, and lack of washability. Despite these challenges, ongoing research and development efforts aim to address these concerns and further enhance the effectiveness and usability of these novel sleep monitoring solutions [9,10].

#### 3.4.1. Bed Structure Devices

Sensors can be attached/integrated to the bed structure for vital signs monitoring.

Feng et al. [70] developed a custom-made ultrasensitive accelerometer, attached to the bed frame, tailored for capturing BCG signals. This sensor marks the first of its kind, designed to extract key features such as RR and HR with high precision and sensitivity. This low-cost sensor has a unique structure comprising a cascade asymmetrical-gapped cantilever, yielding superior resolution compared to conventional accelerometers found in smartphones and smartwatches. Moreover, the sensor enables precise detection of body movements, enhancing its utility for comprehensive sleep monitoring. Beyond its application in beds, Feng and his team emphasize the versatility of this sensor, highlighting its compatibility with other furniture such as chairs and sofas. Accelerometer technology was also employed by Boiko et al. [71]. The team attached a special holder to the bed slats (under the bed mattress), with an accelerometer at one end of a spring steel plastine (hanger). This last component is loose and moves freely with the respiratory and heart movements, which are then detected by the sensor.

Su et al. [72] engineered a bed sensor system designed to monitor BCG signals for cardiovascular assessment, namely, the HR and BP. This system comprises four parallel tubes filled with water, strategically affixed to the bed frame beneath the mattress. Each tube is equipped with a pressure sensor at its end, enabling the precise capture of BCG signals. The synchronized BCG signals collected from these sensors are then used to obtain the HR and the BP values.

F. Li et al. [73] developed, in 2021, a prototype integrated into the bed frame. This system comprises a seismometer, engineered to capture vibration data throughout the sleep cycle. Using advanced machine learning (ML) algorithms, the system seamlessly analyzes the vibration signals to derive essential metrics such as HR and RR, body movements, position changes, and bed occupancy.

Clemente [74] and Song [75] used geophones attached to the bedframe for continuous monitoring of HR, RR, and interbeat interval while sleeping. The first group presented a system called Helena, which employs a very simple algorithm for vital signs calculation, and it also estimates when the user enters and exits the bed [74], while Song’s group presents the BedDot, a monitoring system that uses a more robust calculation algorithm, obtaining better results in the vital parameters estimation. Additionally, they continuously monitor the presence of the user in bed [75,76]. This system is clinically validated and commercialized by the Intelligent Dots company (Peachtree Corners, GA, USA) [77].

#### 3.4.2. Smart Mats

Smart mats are evolving for better sleep monitoring, integrating their available features for vital signs monitoring. These mats can be placed above or below the main bed’s mat or can be the mat itself.

Walsh et al. [78] developed a pressure-based under-mattress mat, composed of optical fibers enclosed in a foam mat. The pressure was estimated by measuring the amount of light received by the detector. When pressure is applied to the mat, the thickness of the foam is reduced, decreasing the light passing between the emitter and the detector. The light signal from each fiber is then converted into an electrical signal to be analyzed by the algorithm to extract the RR, posture changes, and other features. This device is placed beneath the thorax of the subject.

M. Peng et al. [79] designed an intelligent system with 18 piezoelectric sensors, placed below the mattress, for capturing the pressure changes. A channel-selection algorithm identifies the sensor with the strongest signal, and three methods are used to extract physiological data: wavelet analysis, ensemble empirical mode decomposition, and a novel low-complexity dynamic smoothing algorithm. Among them, dynamic smoothing is designed for real-time implementation in low-cost embedded systems. Experimental results demonstrate that the piezoelectric sensors effectively capture biosignals, and dynamic smoothing achieves an accuracy comparable to that of more complex methods while significantly reducing computational and memory demands [76,79].

Wang and his group [80] presented a mattress instrumented with an optical fiber Mach–Zender interferometer for continuous monitoring of human vital signs. This type of sensor measures strain, pressure, and vibration through phase modulation. The mattress is composed of a single-mode fiber placed in the middle of two polyvinyl chloride layers, and a processing system for signal demodulation and features extraction (HR, heart wave’s amplitude, RR, respiratory wave’s amplitude, states of activity: on/off bed and body movements).

M. Laurino et al. [81] developed a pressure-mapping mat, which detects the pressure distribution of the subject lying on the bed. They use pressure signals to analyze the subject’s position, body movements, and RR. The developed system is based on a piezoresistive textile placed between two layers of electrodes (one in columns, and the other in rows). The cross-section between the electrodes’ rows and columns represents a sensing area, where the resistance decreases when the pressure increases. These researchers also distributed three accelerometers over the mat to record the BCG and extract the subject’s HR. This system was then placed on a foam layer below the top cover of the mattress.

Some research groups worked on extracting users’ ECG through clothes while they were sleeping. S. Peng’s group [82] and Resta’s group [83] were able to access the HR and RR through an ECG system based on three electrodes of flexible silver fiber fabric integrated into a mattress surface. Two of the electrodes (active electrodes) are placed under the chest and waist, while the remaining is under the pelvis. Wang et al. [84] applied a total of 44 electrodes fabricated on a flexible printed circuit, which is placed under the mattress cover sheet. This high number of sensors guarantees that the system is adaptable for various body sizes and sleep postures. From the ECG signals, they extract the HR and RR values, as well as the sleeping posture, by applying machine learning algorithms based on neural networks.

**Figure 6:**
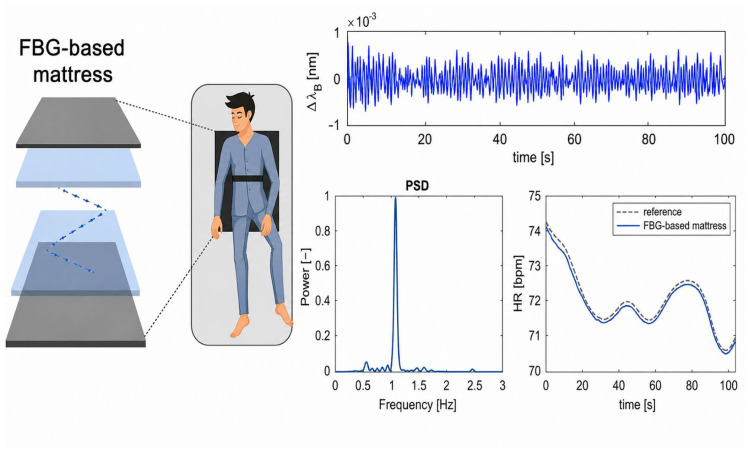
Smart mattress based on FBG technology. Image adapted from ref. [86].

De Tommasi et al. developed a mattress based on FBG sensors for HR and RR monitoring. They incorporated an array of 13 FBG sensors in multiple layers of silicon and studied the feasibility of measuring RR in different sleeping positions [85]. Recently, the same group used the same technology for monitoring HR under different breathing conditions and sleeping postures, as can be seen in Figure 6 [86].

There are several commercially available solutions offering advanced sleep monitoring capabilities:

- The Withings sleep tracking mat, developed by Withings (Issy-les-Moulineaux, France), stands out for its capacity to detect sleep apnea by analyzing RR, HR, and body movement. Positioned discreetly beneath the mattress, this mat utilizes pneumatic and sound sensors to gather data throughout the night. Post-sleep, the accompanying app processes the collected information, providing users with comprehensive insights into their sleep patterns [87,88].
- Emfit (Vaajakoski, Finland) contributes to this field with the Emfit QS, an under-mattress device designed for monitoring HR, RR, and body movements. Utilizing owner-designed qazi-piezoelectric sensors for BCG acquisition, the Emfit QS provides comprehensive sleep data through a subscription service in the user’s phone [87,89].
- ChiliPad by SleepMe (Mooresville, NC, USA) offers an unobtrusive sleep tracking solution designed to be placed discreetly under a mattress topper or sheet. This innovative device monitors various sleep metrics, including HRV and RR, providing users with valuable insights into their sleep quality and overall health. The system consists of a thin, flexible mat placed beneath the bedding, which seamlessly captures sleep data throughout the night. This mat is connected to a processing unit, which collects and analyses the gathered information in real time [90].
- RestOn, from the Sleepace company (Shenzhen, China), is a sleep monitor in a belt shape, which is placed on the mattress, near the thorax. This device tracks the HR, RR, and body movement [91]. This company also has a complete mattress that monitors the sleep of two people at the same time (HR, RR, body movement, etc.). The control box for data analysis is also integrated into the mattress [92]. Like in the pillow, this company does not mention the sensors used in these devices for vital signs monitoring.

#### 3.4.3. Smart Bed Sheets

With the rising of smart textiles, smart bed sheets are becoming available for health monitoring while in bed. Babusiak et al. have introduced an innovative approach to ECG measurement with the development of a contactless bed sheet system. This solution utilizes capacitively coupled textile electrodes, integrating conductive fibers to facilitate non-intrusive ECG monitoring. The bed sheet is equipped with eight squared active electrodes strategically positioned to ensure robust signal acquisition. Additionally, it has two rectangular areas: one acts as a positive electrode, while the other functions as a driven right leg circuit, effectively mitigating noise interference. This prototype has demonstrated promising results in capturing clean ECG signals, laying the groundwork for the accurate calculation of cardiovascular system features [41].

In the realm of smart bedding solutions, Studio 1 Labs (Markham, ON, Canada) offers a notable example with the Bed Sheet Monitor [44]. This innovative device serves multiple purposes, including fall monitoring, pressure ulcer detection, and tracking breathing patterns. Utilizing pressure-sensing fabric technology, it discreetly gathers data throughout the night, offering insights into various aspects of users’ sleep and health. Unlike some competing products, the Studio 1 Labs Bed Sheet Monitor does not feature a dedicated mobile application for data collection and display. However, it offers the convenience of connecting directly to a computer, enabling users to access and analyze the acquired data easily. This flexibility in data retrieval makes it a versatile tool for both personal and clinical use cases [43,44,87].

EightSleep (New York City, NY, USA) [42] offers a sophisticated sleep monitoring system comprising a mattress cover paired with a pod, providing comprehensive sleep tracking and personalized comfort features. The mattress cover is equipped with 36 biometric sensors strategically placed to monitor vital signs, including HR, HRV, and RR, through vibration detection. This extensive sensor array enables detailed insights into sleep quality and overall health metrics. In addition to biometric monitoring, the mattress cover incorporates water pipes for temperature regulation, with connectivity to the pod for precise control of heating or cooling functions. This innovative feature allows users to adjust mattress temperature to their preference, enhancing comfort and promoting better sleep. The pod further enhances the sleep experience with its Autopilot functionality, an advanced algorithm designed to analyze sleep patterns and biometric data in real time. Using this data, Autopilot autonomously adjusts mattress settings such as elevation and temperature to optimize sleep quality throughout the night.

#### 3.4.4. Smart Pillows

Pillows are another essential component for bedroom-based sleep monitoring systems. They usually feature sensing systems that detect changes in pressure and correlate them with HR, RR, and body movements. These systems can be placed outside or inside the pillow.

A research group of S. Li developed a pillow equipped with three temperature sensors and one humidity sensor positioned on the pillow’s surface. The sensors monitor BT, sweat levels, and head positioning. Powered by a lithium battery, this health-sensing system uses a BLE/Zigbee technology for data processing and transfer over Bluetooth to a mobile phone or Wi-Fi, subsequently uploading it to an internet cloud server for analysis [93].

W. Wang et al. [94] introduced InPillow, an innovative intelligent pillow designed to track vital signs, such as RR and HR, as well as body movement during sleep. The InPillow employs a piezoelectric ceramic sensor integrated into a pillow’s cavity to capture the vital signs accurately. A main control board, responsible for data acquisition and processing, a Wi-Fi communication module, and a battery for uninterrupted operation complement this smart sensing system. HR, RR, and body movement are derived from the collected BCG signal data obtained by the piezoelectric sensor.

H. Wang et al. [95] introduced, in 2023, the SleepSense, a smart pillow leveraging a Fiber Bragg Grating (FBG) sensor array to monitor head movements during sleep. The FBG array is ingeniously embedded within silicone buttons, enhancing pressure-sensing capabilities while ensuring system durability. In addition, SleepSense has the capability to monitor RR. This dual functionality showcases the versatility and potential applications of FBG sensing technology in sleep monitoring devices, offering a comprehensive solution for assessing sleep quality and respiratory health.

#### 3.4.5. Wireless Devices

**Figure 7:**
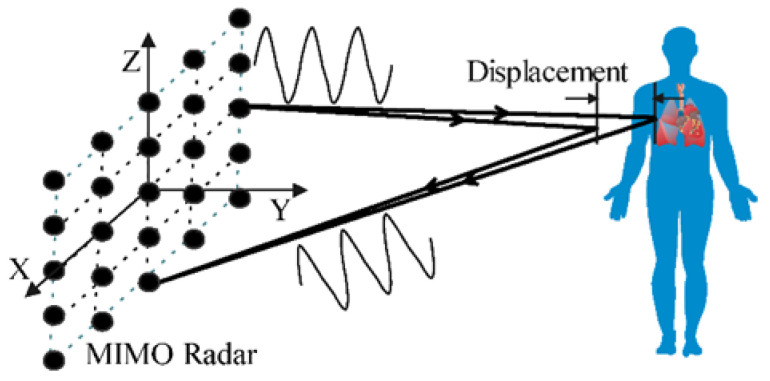
Radar-based systems’ work principle for sleep and health monitoring: Vitalthings Somnofy from Vitalthings [96]; S+ by Resmed [98]; and Sleepiz [99].

Wireless devices can measure sleep from a distance. Some of these examples are the simultaneous use of Wi-Fi for HR and RR detection, radar systems, and film-based devices, which can be installed near the bed or in the bedroom walls, as seen in the example presented in Figure 7.

Vitalthings Somnofy from Vitalthings (Trondheim, Norway) [96] is presented as a radar-based night guard that can be employed in several environments like hospitals, nursing homes, institutions, and homes [97]. This system collects data while the user is in bed, measuring presence, movements, sleep quality, environment factors, and average RR. The system can be placed above the nightstand or can be hung on the wall.

S+ by Resmed (San Diego, CA, USA) [98] is a radar technology designed to detect the upper body’s movement during sleep, monitoring in this way the RR, overall, the body motion, and posture. It emits very low-power radio waves (10.5 GHz) and then detects the echo of the signal, which is modulated by the body movements. This sensor is placed above the nightstand to better detect the movements from the upper body.

Sleepiz (Zurich, Switzerland) [99] is a health and sleep monitoring system based on radar technology, produced and medically certified in Switzerland. It collects breathing patterns and motion, and it is able to detect the micro movements generated by the heartbeats. It integrates an AI algorithm to classify the sleeping patterns, stages, and other important parameters. The Sleepiz can be placed near the bed, like above the nightstand.

### 3.5. Home Office Environment

Another important space in the house where health monitoring can take place is the home office, especially with the recent rise in remote work. Recently, objects in this space, such as office chairs and computer peripherals, have begun to be seen as useful tools for invisibly monitoring health parameters.

#### 3.5.1. Office Chairs

One of the essential pieces of furniture in the office is the chair. Being able to monitor the vital signs and posture of users that seat for long periods of time is of utmost importance.

Yu et al. [100] proposed a new approach, integrating an electromechanical film sensor under the seat of an office chair to measure pressure changes caused by the user’s body movements and heart activity. The study results suggest that the proposed chair can effectively extract accurate HRV information from the BCG data recorded, which is also useful for stress recognition.

In 2025, a capacitive HR measurement system was integrated into an office chair to detect the user’s ECG, using coupled capacitive electrodes placed in the backrest of the chair. These electrodes are made of conductive fabric tape, and two of them are placed at the same height close to the heart, while the reference electrode is designed to be part of the chair’s backrest cover. The electrodes are connected to an active amplifier and a commercial system that processes and analyzes the biosignals. This system was able to measure HR and HRV [101].

H&T company (Shenzhen, China) offers a smart chair solution, with the aim to improve and support the posture of people who sit for long periods of time, including HR and HRV tracking [102]. As for the other products of this company, already presented in this review, they do not refer to the sensors used in this scenario.

#### 3.5.2. Computer Peripherals

Androutsou et al. [103,104] developed a user-friendly system for detecting occupational stress in office employees by integrating physiological sensors (PPG and galvanic skin response) into a standard computer mouse. The system captures HR and skin conductance data wirelessly via a microcontroller and Wi-Fi module embedded in the mouse. An experimental protocol mimicking office stressors validated the device’s effectiveness, highlighting the system’s potential for real-time, unobtrusive stress monitoring in workplace settings and home office [105].

Consumer earphones and earbuds are increasingly incorporating in-ear PPG sensors for HR and SpO_2_ monitoring. These systems are suited to extended home office wear. Adams et al. tested the in-ear sensor cosinuss° One [106], which collects HR values through PPG and BT. This sensor was clinically validated and compared to ECG recordings and demonstrated a small uncertainty of 0.78 bpm [107]. More recently, Google researchers demonstrated audioplethysmography (APG), which uses the ultrasound signal of active noise cancelation earphones to measure HR and HRV without any additional sensors, achieving a 3.21% median HR error across diverse participants [108].

Camera-based monitoring of blink-rate, eye gaze, and head posture, using the front-facing webcam already present in most laptops, has also been explored as a contactless approach to detect cognitive fatigue and mental workload during computer work [109,110].

While the literature on peripheral-embedded sensing remains sparse, this category is rapidly developing as remote and hybrid work become permanent features of daily life. Future systems may enable comprehensive physiological and cognitive monitoring during computer work without requiring users to interact with any dedicated health device.

### 3.6. Technologies Integrative View

**Figure 8:**
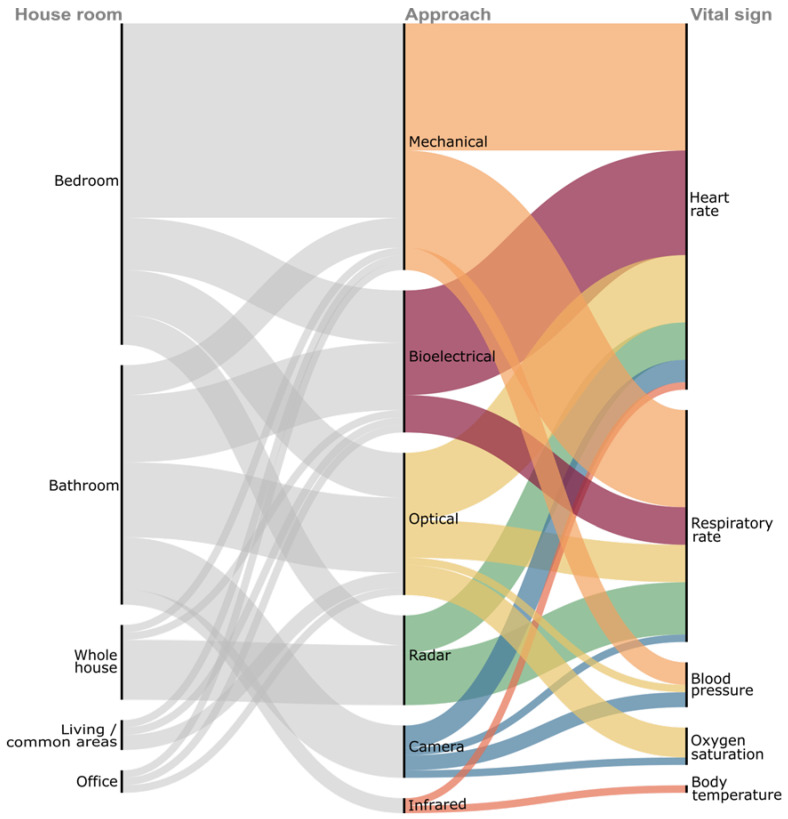
Sankey diagram illustrating the qualitative relationships between home rooms, unobtrusive sensing approaches, and measurable vital signs reported in the literature. Link thickness reflects the prevalence and maturity of reported solutions.

To provide an integrative view of the relationships between home spaces, unobtrusive sensing approaches, and measured vital signs discussed in this section, Figure 8 presents a qualitative synthesis using a Sankey diagram. This representation maps the connections between home rooms, sensing approaches, and physiological parameters reported in this review.

From this qualitative synthesis, it is apparent that unobtrusive health monitoring solutions are more frequently reported in bedrooms and bathrooms, where prolonged occupancy and routine activities enable continuous or repeated physiological sensing during the day. Mechanical and bioelectrical approaches are predominant in these environments, particularly for HR and RR monitoring, while optical and camera-based approaches show broader applicability in shared living spaces. The figure also shows the coexistence of multiple sensing technologies within the same room, highlighting the lack of a unique correspondence between room, sensing approach, and monitoring vital signs.

This pattern of overlapping associations reflects a fundamental characteristic of multimodal smart home systems, where heterogeneous sensing modalities operate concurrently to provide complementary and redundant measurements of the same physiological parameter. Such redundancy enhances robustness and reliability, allowing the system to maintain monitoring performance despite sensor noise, occlusion, or temporary device failure [111].

## 4. Unobtrusive Sensing Methods: Strengths, Limitations, and Research Gaps

### 4.1. Technical Strengths and Capabilities of Unobtrusive Sensing at Home

Unobtrusive home-based sensing technologies offer a powerful pathway toward invisible and personalized healthcare. Their main strength lies in their ability to integrate seamlessly into daily life, enabling continuous monitoring without disrupting established routines. Beyond capturing vital signs, these systems also collect contextual information regarding environmental conditions and user interactions, allowing for a more holistic assessment of well-being. Certain technologies can distinguish between multiple individuals within the same space, tracking movements and associating physiological measurements with specific users, which is fundamental for personalized monitoring strategies.

The diversity of sensing approaches, including camera-based, radar-based, mechanical, bioelectrical, and optical technologies, enables flexible integration into furniture, appliances, and home infrastructure. This diversity of unobtrusive systems enhances the robustness of physiological parameters monitoring by allowing complementary measurements across different house areas. A comparative summary of these modalities, highlighting their strengths, limitations, and smart home applications, is presented in Table 1.

### 4.2. Technical Limitations and Robustness Challenges

Despite their potential, unobtrusive sensing technologies face several technical, ethical, and user-related limitations. Unlike controlled clinical environments, domestic settings introduce substantial variability that can compromise measurement reliability. Motion artifacts, temperature fluctuations, illumination changes, and electromagnetic interference are common sources of signal degradation. In addition, many sensing modalities are affected by signal attenuation, environmental noise, and long-term physical degradation, as summarized in Table 1.

The core challenge of unobtrusive health monitoring lies in achieving adequate performance without compromising user convenience. Sensors embedded in home infrastructure, appliances, furniture, and accessories must provide precise and stable measurements while remaining minimally intrusive and operating under dynamic, uncontrolled conditions. Advanced signal processing and artificial-intelligence techniques have significantly improved data extraction and artifact mitigation; however, robust operation across heterogeneous real-world environments remains an open research challenge [112].

Beyond short-term performance, sustained robustness depends on reliable system operation over extended deployment periods. Sensors may be repositioned, partially obstructed, or exposed to wear, calibration drift, and inconsistent maintenance. Changes in household routines, seasonal conditions, and user behavior further alter operating conditions in ways rarely captured in short-term validation studies. Ensuring dependable long-term monitoring, therefore, requires self-calibration mechanisms, fault detection, and adaptive signal processing strategies capable of maintaining performance under evolving environmental and behavioral conditions. Longitudinal field studies spanning weeks to months are essential to establish sensing accuracy, operational stability, and user acceptance in real-world preventive health applications [112].

### 4.3. Multimodal Sensing and Data Fusion

Multimodal sensing has emerged as an effective strategy for improving the robustness and reliability of unobtrusive health monitoring in private domestic environments, as demonstrated in Figure 8. Individual sensing modalities exhibit modality-specific limitations, and the integration of complementary sensors helps mitigate these weaknesses by leveraging redundancy and contextual information across modalities.

A review from Jonh et al. [111] presents several studies which consistently show that multimodal fusion improves measurement accuracy, reduces false positive rates, and increases robustness under uncontrolled conditions when compared with unimodal approaches. These improvements are especially relevant for long-term, unsupervised home monitoring, where environmental variability and user behavior can significantly degrade single-sensor performance.

A representative example of multimodal integration is provided by smart toilet systems, which combine bioelectrical, optical, and mechanical sensing. This multimodal configuration enables the simultaneous monitoring of parameters such as HR and HRV, using different types of sensors, offering a more comprehensive and reliable assessment than any single sensing modality alone [36,66]. Empirical evaluations suggest that multimodal smart toilet platforms achieve improved robustness under real-world conditions, maintaining consistent performance despite variability in user positioning, contact quality, and environmental factors. Compared with unimodal configurations, the fusion of complementary signals supports more stable longitudinal measurements and enhances the detection of deviations from individual baselines, a key requirement for precision health applications [36].

Multimodal systems typically employ different data fusion architectures depending on application requirements and system constraints. Early-level fusion combines raw or minimally processed signals and can deliver low-level correlations, but it requires high synchronization, good signal quality, and higher computational resources. Feature-level fusion is another modality where the features from each sensing approach are aggregated and then used in ML algorithms. Decision-level fusion is another architecture possibility, where the individual sensing systems provide their own prediction, and the monitoring system combines them through voting or confidence weighting, providing greater modularity and fault tolerance, particularly in home deployments where individual sensors may fail or degrade [111,113].

### 4.4. Clinical Translation, Usability, and System-Level Challenges

A critical limitation that emerges across the reviewed literature is the substantial gap between technical proof-of-concept demonstrations and clinically validated, deployment-ready systems. Many of the reported systems in this review have been evaluated in controlled laboratory conditions using small, healthy volunteer cohorts that do not reflect the diversity of real-world patient populations in terms of age, body composition, comorbidities, and mobility. Validation studies conducted over hours or days under supervised conditions are often insufficient to establish the reliability of systems intended for long-term, unsupervised home use. Furthermore, few studies include rigorous benchmarking against clinical gold standards, such as polysomnography for sleep assessment, under real-world conditions. Regulatory and ethical pathways for home-based continuous monitoring devices remain complex, and only a small number of the reviewed systems have achieved regulatory clearance (e.g., the Heart Seat by Casana (Rochester, NY, USA), FDA-cleared for HR and SpO_2_ monitoring [33,35]). Bridging this translational gap will require larger-scale clinical studies with diverse populations, standardized evaluation protocols, and closer collaboration between technology developers and clinical partners.

This translational gap is also reflected in the development stage (DS) of the reviewed systems. As summarized in Appendix A (Table A1), most unobtrusive home monitoring solutions remain at early to intermediate DS, corresponding to laboratory prototypes or pilot validations, while only a limited number of systems have reached clinical study, commercialization, or regulatory clearance.

At present, most unobtrusive home monitoring systems primarily provide physiological trend monitoring and anomaly detection rather than autonomous clinical decision support. Clinical interpretation typically remains in the hands of healthcare professionals, with system outputs serving as decision-support inputs rather than diagnostic conclusions.

User adherence and acceptability further complicate the implementation of non-intrusive health technologies. Designed to be used in everyday life, these devices rely on consistent use to be effective. Users may change their behavior because they know they are being monitored or might become less attentive to maintaining their devices over time. This can lead to gaps or inaccuracies in the collected data. To address this, it is important to design systems that encourage regular use and promote healthy habits without making users overly dependent on technology. Understanding behavioral psychology can help create features that motivate users to follow health recommendations and stay actively involved in managing their health [112,114].

On the other hand, home health monitoring often involves multiple devices, each capturing different health metrics under various conditions. Combining all these data sources into coherent unified information requires sophisticated data fusion techniques and state-of-the-art knowledge representation methods. The goal is to provide clear, useful insights while avoiding information overload. Ensuring that data integration is both accurate and clinically relevant for users and healthcare providers is a significant challenge that must be taken into account [112,115].

One of the major challenges in continuous health monitoring is the risk of alert fatigue and over-surveillance. While continuous and unobtrusive monitoring offers significant potential for early detection and preventive healthcare, it also introduces the risk of alert fatigue and over-surveillance if not carefully designed. Frequent or non-actionable alerts can lead to desensitization, reduced responsiveness, and increased workload to healthcare professionals, ultimately affecting patient safety and system trust. Excessive false alarms may also create skepticism toward monitoring systems and reduce adherence, a phenomenon commonly described as the “cry wolf” effect [116].

To mitigate these risks, modern precision health frameworks emphasize the importance of delivering actionable information selectively and intelligently, rather than maximizing data collection alone [117]. Strategies such as adaptive alarm thresholds, multimodal sensing, predictive analytics, and user-centered interface design can help prioritize clinically relevant events while minimizing unnecessary notifications. Ultimately, the effectiveness of unobtrusive monitoring systems depends not only on sensing accuracy but also on designing systems that provide meaningful information without overwhelming users, caregivers, or clinicians [116].

### 4.5. Privacy-Preserving Technical Approaches for Unobtrusive Home Monitoring

Privacy protection in unobtrusive home monitoring systems must be addressed not only through ethical protocols and governance policies but also at the level of technical system design. Privacy-preserving architectures aim to reduce unnecessary data exposure while maintaining sufficient analytical performance for health monitoring applications. One foundational approach is on-device processing, enabled by recent advances in embedded microprocessors and edge AI capabilities. By performing signal processing and inference locally, sensitive raw data can be processed at the source and only high-level features or alerts need to be transmitted, thereby reducing privacy risks associated with data transfer and centralized storage [118,119].

Closely related to on-device processing is the use of edge AI, where ML models are deployed at the network edge rather than in remote cloud infrastructures. Edge-based inference offers advantages in latency, robustness, and privacy, particularly in domestic environments where continuous connectivity cannot be assumed. In addition to limiting data exposure, edge AI can support faster detection of health-relevant events and enable autonomous operation during network disruptions, supporting both user safety and system resilience [118,120,121].

For applications requiring collaborative learning across multiple monitoring devices, federated learning (FL) has emerged as a promising privacy-preserving solution. In FL architectures, models are trained collaboratively by sharing model updates rather than raw data, allowing learning from distributed datasets while keeping personal information locally stored. This approach is particularly relevant for unobtrusive home monitoring, where large-scale deployment could otherwise lead to the centralized accumulation of highly sensitive longitudinal health data. However, FL introduces its own technical challenges, including communication overhead, model heterogeneity, and vulnerability to inference attacks, highlighting the need for careful system design and complementary security mechanisms [118,122].

Another important principle is data minimization, which intends to limit data collection, retention, and resolution to what is strictly necessary for the intended health purpose. In unobtrusive monitoring, this may involve selective feature extraction, temporal down-sampling, or discarding raw sensor streams after local processing. Data minimization decreases the attack probability and helps mitigate risks related to unintended secondary data use [123]. However, excessive minimization may compromise clinical utility if relevant contextual or longitudinal information is deleted, reinforcing the need for balanced, application-specific design choices.

### 4.6. Ethical, Privacy, and Governance Implications of Unobtrusive Home Monitoring

Unobtrusive health monitoring technologies raise fundamental ethical and societal challenges precisely because they work continuously within private domestic spaces and often collect data passively, with minimal user awareness. While such systems enable longitudinal data acquisition that is key to precision health, extensive data collection in everyday environments may also be perceived as intrusive or reminiscent of domestic surveillance if not carefully designed and governed. Previous work in precision health has emphasized that public trust, privacy protection, and data security are prerequisites for sustained user participation and system effectiveness [124].

One of the main ethical requirements for this type of monitoring system is privacy-preserving design. Precision health technologies must reconcile the collection of accurate, continuous health data with strict data minimization and protection principles. Privacy-by-design approaches, including on-device processing, selective data retention, encryption at rest and in transit, and privacy-preserving analytics, are essential to prevent unnecessary exposure of sensitive personal information [124].

Informed consent represents a particularly complex challenge in passive and continuous monitoring scenarios. Unlike wearable devices, unobtrusive sensors embedded in furniture, appliances, or infrastructure may operate without explicit user interaction. As discussed in the context of smart toilets, consent models based only on one-time consent are often inadequate when monitoring is continuous, difficult to avoid, or shared among multiple household members [36]. Adaptive and ongoing consent models, transparency about what data are collected and how they are used, and meaningful user control over data access and system activation are therefore critical to preserve users’ autonomy in their home.

There are closely related concerns regarding data governance and secondary use of personal health data. Continuous in-home monitoring generates large amounts of sensitive data that may be attractive for secondary uses beyond the original health purposes, including commercial analytics or population-level research. Prior studies in precision health refer that unclear data ownership and poorly defined governance frameworks affect trust and may discourage users’ participation [124]. Robust governance models should therefore clearly assign data ownership to users, define permissible secondary uses, enforce auditability, and allow permanent deletion of data upon request [125].

The risk of domestic surveillance and over-monitoring is another ethical issue that emerges across unobtrusive sensing applications. The used sensors may unintentionally capture behavioral patterns, routines, or intimate activities beyond strict clinical relevance. As pointed out in smart toilet monitoring, even when data are anonymized, certain forms of sensing, such as imaging or behavioral inference, may still be perceived as intrusive, particularly in highly private contexts like bathrooms and bedrooms [36]. These concerns are consistent with broader evidence that anonymization alone is insufficient to mitigate privacy harm when high-resolution or linkable data streams are collected continuously [126]. Ethical deployment, therefore, requires careful consideration of sensor placement, modality selection, and the proportionality between sensing granularity and clinical benefit.

Taken together, these ethical, privacy, and governance considerations are essential for translating unobtrusive home monitoring technologies from experimental settings into trustworthy, scalable Healthcare 5.0 solutions. Addressing these issues alongside technical performance is critical to achieve long-term adoption, public trust, and societal benefit.

### 4.7. Societal, Economic, and Accessibility Considerations

Cost and accessibility are also crucial factors: high costs can limit access to technology. For these technologies to be impactful, they must be affordable and accessible for a diverse population. Cost reduction is a crucial challenge, critical to create the conditions for massification of these technologies. Designing and developing inclusive solutions is a major challenge, augmented by the inherent diversity of inhabitants regarding age, size, technological competencies, and health conditions, to name a few. Being that many of the reviewed technologies are prototypes or in initial commercialization stages, not much work has been done to make them widely adopted, a major challenge for the future [112,114].

The future of well-being through invisible health technology offers significant potential but comes with a set of complex challenges. Addressing these challenges requires a concerted effort from technologists, healthcare professionals, vendors, regulators, and users. By overcoming these hurdles, invisible health technologies at home have great potential to help revolutionize healthcare, making it more personalized, preventive, proactive, and accessible to all.

## 5. Opportunities and Future Directions

Healthcare is changing day by day. This review highlights the growing focus of health monitoring at home, from wearables to invisibles, consequently changing the way society self-monitors health parameters. With these developments, healthcare monitoring is becoming more intuitive and user-friendly.

### 5.1. Technical Directions

Unobtrusive technologies for health tracking are more than simple vital sign trackers, as they also gather information about the environment and the way that the users interact with it. Additionally, some of them are able to distinguish between different people in the same room, tracking their movements and monitoring their vital signs, providing identification of the person and the context, essential for personalization [127].

These technologies show huge potential but also leave room for improvement since many of them are still being developed. Wireless sensing (e.g., radio-waves and Wi-Fi) is paving the way for these developments, for a continuous tracking of people’s movements, activities, and health parameters through all the rooms at home. To complement these sensing systems, other systems implemented in the house’s furniture and accessories can communicate through a network, understanding the way people interact with these technologies [128].

The evolution in sensing, combined with the current era of IoT, where multiple devices are able to communicate with each other and/or with a central hub, establishes a fertile ground for the growth of in-house complex data acquisition infrastructures. By gathering and correlating the information from multiple sources, it is possible to extract important health parameters and contribute to an overall and informed improvement of well-being [127].

Most of the technologies presented in this review require a power supply to operate. Being able to use self-powered sensors can be a reliable solution to eliminate wired connections. Some of these examples include triboelectric [129], piezoelectric, and pyroelectric nanogenerators, solar collectors, and biofuel cells [112]. Another solution is to use wireless power transfer for charging these devices. Available technology achieves power transfer using far-field methods, such as acoustic, optical (laser power transfer), and microwave energy as carriers, or near-field methods, including non-radiative electromagnetic fields, primarily through inductive or capacitive antenna coupling. Further developments of these technologies will allow the development of devices that can run for several years without any power supply-related maintenance [130].

Traditionally, the extraction of health-related information from sensor data has relied on training ML models either offline or in the cloud. However, recent advances in microprocessor technology, used in most sensing devices, have enabled the integration of pre-trained ML models directly into their firmware. This local (on-device) processing adds an extra layer of capabilities for decision-taking, allowing these devices to indicate health abnormalities more quickly and autonomously, significantly reducing the need for human intervention [127].

### 5.2. Clinical Directions

With the advent of a new golden era of AI, powered by fast GPUs, deep learning, and large datasets, the data gathered from several home appliances can provide rich insights about the inhabitants. This includes complex health parameters analysis, detection of physiological deviations, understanding how people interact at home, and identification of behavior changes. Such information can be quickly reported to the nearby hospital and/or caregivers, enabling timely intervention if needed [127].

One of the most important challenges lies in translating raw sensor data into validated, actionable health indicators that integrate seamlessly with existing care pathways. Rather than replacing traditional clinical assessments, unobtrusive monitoring technologies are likely to function as complementary tools, supporting longitudinal observation and personalized health management across diverse populations.

Across sensing modalities and domestic applications, DS analysis highlights a clear imbalance between technical innovation and clinical maturation. While numerous solutions demonstrate feasibility, comparatively few have progressed to longitudinal clinical evaluation or regulatory approval, emphasizing the need to focus future research efforts on validation, interoperability, and integration into healthcare workflows.

### 5.3. Ethical and Privacy Directions

Ethical considerations become increasingly central to the future development of unobtrusive monitoring systems. Continuous and passive data collection in private spaces raises concerns related to privacy, autonomy, and governance. Ensuring that users retain meaningful control over data generation, processing, and sharing is essential for ethical deployment [124].

Future systems must therefore prioritize privacy-preserving architectures, including local data processing, selective retention, and secure communication mechanisms. Additionally, governance frameworks should ensure that personal health data remain under user ownership, can be shared selectively with trusted parties such as healthcare professionals, and can be permanently deleted upon request. Addressing these issues alongside technical developments is critical for sustaining trust and long-term adoption of these technologies [124,125].

### 5.4. Societal Directions

Looking beyond individual sensing modalities, the integration of unobtrusive home monitoring with advances in AI and cloud computing enables a transformative paradigm: the development of a DigitalMe, which is a continuously updated digital twin that integrates multimodal longitudinal data streams into a comprehensive, individualized model of health [131]. Rather than relying on isolated measurements of specific physiological parameters, unobtrusive home sensing enables the continuous collection of heterogeneous data (HR, RR, sleep monitoring, activity tracking, and behavioral interactions), which can be fused over time to build a dynamic representation of an individual’s health trajectory.

This longitudinal and person-specific model has the potential to detect deviations from personal baselines significantly earlier than population-level reference values, enabling truly personalized and predictive healthcare. Within the context of smart cities and broader digital health ecosystems, the DigitalMe concept positions the home as a critical node within an interconnected health infrastructure, linking personal monitoring data with clinical systems, environmental sensing networks, and community-level health surveillance [131].

Achieving this paradigm will require progress not only in sensing technologies and AI algorithms but also in interoperability standards, long-term data management, and privacy-preserving system architectures. Addressing these technical and governance challenges represents a critical and timely frontier for the field.

## 6. Conclusions

The emergence of unobtrusive health monitoring technologies at home represents a revolution in healthcare, enabling continued and non-intrusive monitoring of individuals’ health through integration of these technologies in home environments and objects. The potential of this approach in advancing healthcare is evident: it enables a more patient-centered model, provides fast insights into various health metrics, facilitates early detection of potential issues, promotes preventive care, and aligns with the Health 5.0 vision, making healthcare more personalized, accessible, and proactive.

Key findings in this review are the wide range of applications of unobtrusive health technologies across home spaces, from sensor-embedded sofas and cushions to smart mirrors and toilets, each offering valuable health insights without disrupting individuals’ daily routines.

Nonetheless, the widespread use of these technologies presents a series of complex challenges. Ensuring the accuracy of measurements, developing affordable, accessible, and discreet non-intrusive systems, addressing data security and privacy concerns, promoting user engagement, and providing a good user experience are all critical factors that should be considered to achieve high acceptance of these technologies in the home setting.

Invisible health technologies have significant potential for improvement, as many of them are still in the early stages of development. Powered by advancements in wireless sensing and IoT infrastructure, these systems can gather and correlate data from multiple sources to provide important health insights and support proactive well-being management. AI enhances these capabilities, enabling the detection of health problems, behavioral analysis, and supporting more informed decision-making. On the other hand, the integration of self-powered sensors and wireless power transfer offers the potential for long-term, maintenance-free operation. For these technologies to achieve their full potential, robust and secure communication infrastructures are essential to connect home-generated information to healthcare providers, ensuring user privacy and control over personal health data. As these technologies continue to develop, they hold significant promise in improving health monitoring and overall well-being.

## Figures and Tables

**Table 1:** Comparison between vital sign measurement approaches and their application in a smart-home environment.

Sensing Approach	Strengths	Limitations	Smart Home Application
Camera-based (RGB, NIR, FIR)	RGB enables multi-parameter extraction (HR, RR, SpO_2_, BP) NIR/FIR operate in low-light or darkness FIR reduces identifiability (improved privacy vs. RGB)	Sensitivity to illumination changes (RGB) Motion artifacts degrade signal quality NIR: lower hemoglobin absorption, reduced SNR FIR: high-cost Privacy and data security concerns	Smart mirrors
Laser-based	Contactless measurement Partial penetration through clothing or thin materials High sensitivity to micro-vibrations	Strongly affected by body motion Thermal distortion (sweating, airflow) Limited robustness in uncontrolled environments	
Radar-based (CW, UWB, FMCW)	Fully contactless Operates through obstacles (especially UWB) CW: simple architecture, low-power UWB: high spatial resolution FMCW: multi-target discrimination, energy-efficient	Susceptible to interference (other devices, moving subjects) UWB: signal attenuation over distance FMCW: requires advanced signal processing Algorithmic complexity for vital sign extraction	Whole-house monitoring, in-wall systems, bedroom radar devices
Infrared sensors (PIR, thermal)	Low-cost Low-power Wide detection area Simple deployment	Low spatial resolution Sensitive to ambient temperature changes Requires clear line of sight Limited capability for precise physiological estimation	Smart mirrors
Mechanical sensors (capacitive, piezoelectric, piezoresistive, triboelectric)	Easily embedded in furniture Suitable for BCG/SCG acquisition Some are self-powered (piezoelectric, triboelectric) No direct skin contact required	Sensitive to environmental noise (vibration, humidity, temperature) Mechanical fatigue over time Motion artifacts Posture-dependent signal quality	Floor tiles, cushions, bathmats, toilets, bed structure, smart mats, smart bed sheet, pillows, office chairs
Bioelectrical sensors	Clinically validated signals High diagnostic relevance Direct electrophysiological measurement	Electrode durability Susceptible to electromagnetic interference	Floor tiles, chairs, bathtubs, toilets, smart mats, smart bed sheets, office chairs
Optical sensors	Multi-parameter monitoring (HR, RR, SpO_2_) Fiber optics enable strain/pressure sensing Electrically safe	Motion artifacts Ambient light interference Contact PPG requires skin proximity Fiber fragility	Cushions, toilets, smart mats, pillows, computer peripherals

## Data Availability

Data sharing is not applicable.

## Abbreviations

The following abbreviations are used in this manuscript:

AI	Artificial intelligence
IoT	Internet of things
IR	Infrared
ECG	Electrocardiography
BCG	Balistocardiography
PPG	Photoplethysmography
RGB	Red-green-blue
NIR	Near-infrared
FIR	Far-infrared
HR	Heart rate
RR	Respiratory rate
SpO_2_	Oxygen saturation
BP	Blood pressure
SNR	Signal-to-noise ratio
CW	Continuous wave
UWB	Ultra-wideband
FMCW	Frequency-modulated continuous wave
PIR	Pyroelectric infrared
SCG	Seismocardiography
EEG	Electroencephalography
EMG	Electromyography
MIMO	Multiple-input multiple-output
RIS	Reconfigurable intelligent surface
HRV	Heart rate variability
BT	Body temperature
EST	Exercise stress test
CVDs	Cardiovascular diseases
PSG	Polysomnography
ML	Machine learning
FBG	Fiber Bragg grating
APG	Audioplethysmography
FL	Federated learning
DS	Development stage

## Appendix A

**Table A1:** Summary of the reviewed technologies.

House Room	Object	Name/Company	Vital Signs (Method)	Sensor(s)	DS	Year	Ref.
General household monitoring	Floor tiles	--	HR (ECG, BCG)	Load cells, Stainless steel electrodes	Pilot validation	2018	[31]
	Wireless devices	--	HR, RR	UWB radar—MIMO imaging radar	Prototype	2021	[32]
		VitalHub	HR, RR	UWB radar combined with depth camera	Prototype	2022	[18]
		--	HR, RR	RIS	Prototype	2023	[47]
		Cardi/o/Advanced TeleSensors (Austin, TX, USA)	HR, RR	Low-power radio waves	Commercialized	2025	[46,48]
Living room and common areas	Cushions	--	HR (BCG)	Strain-gauge load cells	Prototype	2021	[49,50]
		--	HR, RR (BCG)	Microbend fiber sensor	Prototype	2019	[52]
	Sofas	--/H&T (Shenzhen, China)	HR, RR	--	Commercialized	--	[53]
	Chair	--	HR, HRV (ECG)	Capacitive coupling electrodes	Pilot validation	2025	[51]
Bathroom	Mirrors	Medical Mirror	HR	Thermal camera	Prototype	2018	[58,60]
		Anura MagicMirror/NuraLogix (Toronto, ON, Canada)	HR, RR, BT, BP, HRV	Camera	Commercialized and Regulatory clearance	2024	[59]
		Artemis/CareOS (Neuilly-sur-Seine, France)	HR, RR, BP, SpO_2_	RGB camera, IR temperature sensor, UV light	Commercialized	2019	[55,56]
		Themis/CareOS (Neuilly-sur-Seine, France)			Commercialized	2021	
	Bathmats	Bbalance/Baracoda (France)	Footprint, posture, weight, balance, body composition	Pressure sensors	Commercialized	2020	[61]
	Bathtubs	--	HR, RR (ECG)	Capacitive coupling electrodes	Prototype	2017	[63]
		--	HR, HRV (ECG)	Electrodes	Prototype	2022	[62]
	Smart toilets	Heart Seat/Casana (Rochester, NY, USA)	HR, BP, SpO_2_ (ECG, BCG, PPG)	Stainless steel electrodes, reflective optical sensor, load cells	Commercialized and Regulatory clearance	2023	[33,35]
		--/OLI (Aveiro, Portugal)	HR (ECG)	Dry polymer electrodes	Pilot validation	2021	[39]
		--	BT, SpO_2_ (PPG)	Temperature sensor, optical sensor		2022	[65]
		Çava Seat/Çava Health (Irvine, CA, USA)	HR, HRV, SpO_2_, BP, BT (ECG, PPG)	Capacitive electrodes, optical sensors, bioimpedance sensors, surface temperatures sensors	Commercialized	2019	[34]
		Synsol/Geometry Healthcare (Beijing, China)	HR, RR (ECG)	--	Commercialized	2021	[40]
		One Planet/One Planet Research Center (Nijmegen and Wageningen, The Netherlands)	HR, RR, SpO_2_, BP, BT (ECG, PPG)	Electrodes, temperature sensor, optical sensor, load cells	Commercialized	2023	[38,68]
		Haro/Hamberger Medical (Stephanskirchen, Germany)	HR, HRV (ECG)	6–lead ECG electrodes	Commercialized and Regulatory clearance	--	[37]
Bedroomintell	Bed structure	--	HR, RR (BCG)	Accelerometer	Prototype	2017	[70]
		--	HR, RR	Accelerometer	Prototype	2023	[71]
		--	HR, BP (BCG)	Hydraulic pressure sensors	Pilot validation	2019	[72]
		--	HR, RR	Seismometer	Pilot validation	2021	[73]
		Helena/--	HR, RR, HRV	Geophone	Pilot validation	2020	[74]
		BedDot/Intelligent Dots (Peachtree Corners, GA, USA)	HR, RR, HRV	Geophone	Commercialized	2024	[75,77]
	Mats	--	RR	Optical fiber	Prototype	2017	[78]
		--	HR, RR	Piezoelectric sensors	Prototype	2019	[79]
		--	HR, RR	Optical fiber Mach-Zender interferometer	Pilot validation	2020	[80]
		--	RR	Piezoresistive textile and electrodes	Prototype	2020	[81]
		--	HR, RR (ECG)	Electrodes of flexible silver fabric	Prototype	2021	[82]
		--	HR, RR (ECG)	Electrodes of flexible silver fabric	Prototype	2024	[83]
		--	HR, RR (ECG)	Capacitive coupled electrodes	Pilot validation	2023	[84]
		--	HR, RR	FBG sensors	Prototype	2023	[85,86]
		Withings Sleep Tracking Mat/Withings (Issy-les-Moulineaux, France)	HR, RR	Pneumatic sensors, sound sensors	Commercialized and Regulatory clearance	--	[88]
		Emfit QS/Emfit (Vaajakoski, Finland)	HR, RR (BCG)	Qazi-piezoelectric sensors	Commercialized	--	[89]
		ChiliPad/SleepMe (Mooresville, NC, USA)	HRV, RR	--	Commercialized	--	[90]
		RestOn/Sleepace (Shenzhen, China)	HR, RR	--	Commercialized	--	[91]
	Bed sheets	--	HR (ECG)	Capacitive coupled textile electrodes	Prototype	2019	[41]
		Bed Sheet Monitor/Studio 1 Labs (Markham, ON, Canada)	RR	Pressure sensors	Clinical study	--	[44]
		EightSleep/-- (New York City, NY, USA)	HR, HRV, RR	Biometric sensors	Commercialized	--	[42]
	Pillows	--	BT	Temperature sensors, humidity sensors	Prototype	2018	[93]
		InPillow/--	HR, RR (BCG)	Piezoelectric ceramic sensor	Pilot validation	2020	[94]
		SleepSense/--	RR	FBG sensor	Prototype	2023	[95]
	Wireless devices	Vitalthings Somnofy/Vitalthings (Trondheim, Norway)	RR	Radar based	Commercialized and Regulatory clearance	--	[96]
		S+/ResMed (San Diego, CA, USA)	RR	Low-power radiowaves	Commercialized	--	[98]
		Sleepiz/-- (Zurich, Switzerland)	HR, RR	Radar based	Commercialized and Regulatory clearance	--	[99]
Home office	Office chairs	--	HR, HRV (BCG)	EMFi sensor	Pilot validation	2019	[100]
		--	HR, HRV (ECG)	Capacitive coupled electrodes	Prototype	2025	[101]
		--/H&T (Shenzhen, China)	HR, HRV	--	Commercialized	--	[102]
	Computer peripherals	--	HR (PPG)	Optical sensors, galvanic skin sensors	Pilot validation	2023	[103,104]
		Cosinuss° One/Corpuls (Kaufering, Germany)	HR (PPG), BT	Optical sensors	Commercialized and Regulatory clearance	2022	[106,107]
		--/Google	HR (APG)	Ultrasounds	Pilot validation	2023	[108]
